# Computational and Biological Comparisons of Plant Steroids as Modulators of Inflammation through Interacting with Glucocorticoid Receptor

**DOI:** 10.1155/2019/3041438

**Published:** 2019-05-27

**Authors:** Mohamed A. Morsy, Snehal S. Patel, Azza A. K. El-Sheikh, Jignasa K. Savjani, Anroop B. Nair, Jigar N. Shah, Katharigatta N. Venugopala

**Affiliations:** ^1^Department of Pharmaceutical Sciences, College of Clinical Pharmacy, King Faisal University, Al-Ahsa 31982, Saudi Arabia; ^2^Department of Pharmacology, Faculty of Medicine, Minia University, El-Minia 61511, Egypt; ^3^Department of Pharmacology, Institute of Pharmacy, Nirma University, Ahmedabad, Gujarat 382 481, India; ^4^Basic Health Sciences Department, Faculty of Medicine, Princess Nourah bint Abdulrahman University, Riyadh 11671, Saudi Arabia; ^5^Department of Pharmaceutical Chemistry, Institute of Pharmacy, Nirma University, Ahmedabad, Gujarat 382 481, India; ^6^Department of Pharmaceutics, Institute of Pharmacy, Nirma University, Ahmedabad, Gujarat 382 481, India; ^7^Department of Biotechnology and Food Technology, Durban University of Technology, Durban 4000, South Africa

## Abstract

Despite the usefulness of glucocorticoids, they may cause hazardous side effects that limit their use. Searching for compounds that are as equally efficient as glucocorticoids, but with less side effects, the current study compared plant steroids, namely, glycyrrhetinic acid, guggulsterone, boswellic acid, withaferin A, and diosgenin with the classical glucocorticoid, fluticasone. This was approached both *in silico* using molecular docking against glucocorticoid receptor (GR) and *in vivo* in two different animal models. All tested compounds interacted with GR, but only boswellic acid and withaferin A showed docking results comparable to fluticasone, as well as similar *in vivo* anti-inflammatory effects, by significantly decreasing serum levels of interleukin-6 and tumor necrosis factor-*α* in cotton pellet-induced granuloma in rats. In addition, both compounds significantly decreased the percent of change in ear weight in croton oil-induced ear edema in mice and the granuloma weight in cotton pellet-induced granuloma in rats, to levels comparable to that of fluticasone. Both boswellic acid and withaferin A had no effect on adrenal index, but only withaferin A significantly increased the thymus index. In conclusion, boswellic acid may have comparable anti-inflammatory effects to fluticasone with fewer side effects.

## 1. Introduction

Glucocorticoids are indispensable pharmacological products employed in the treatment of various diseases, including cancers, autoimmune disorders, bronchial asthma, anaphylaxis, and adrenal insufficiency [1]. These drugs' usage in treatment of diverse unrelated diseases arises from the abundance of their receptor, the glucocorticoid receptor (GR). The GR is encoded by a nuclear receptor, NR3C1 gene, and expressed in different body tissues, where it mediates a wide variety of ubiquitous functions [2], as the GRs are nuclear receptors capable of regulating various biological functions, including metabolism, body growth, and inflammation. Many pharmacological products were developed to take advantage of GR as a therapeutic target, including fluticasone that is considered a highly selective substrate of GR, with long duration of action reaching up to twenty-four hours due to its receptor fast association/slow dissociation properties [3].

Unfortunately, despite the high therapeutic efficacy of glucocorticoids in treating different disorders, they might cause several side effects, including the increased susceptibility to infections [4] and adrenal insufficiency [5]. Such harmful side effects limited the use of glucocorticoids. Interestingly, several compounds from plant origins have been suggested to possess steroid-like activity, probably due to their chemical resemblance with glucocorticoids [6]. These plant steroids include glycyrrhetinic acid, an active ingredient of licorice that has been implicated in the treatment of rheumatoid arthritis [7]. Guggulsterone is also a plant steroid that is the major active constituent of the gum guggul plant, whose extract, guggulipid, has shown beneficial effects when applied topically for treatment of acne [8]. Another plant steroid is boswellic acid, which is the active ingredient of the Indian frankincense *Boswellia serrata*, a type of aromatic gum resin that has been patented for promoting the healing of burns, as well as treatment of pain, rheumatoid arthritis, and degenerative musculoskeletal diseases [9]. Withaferin A, the active ingredient of *Withania somnifera* commonly known as ashwagandha or Indian ginseng, is also a plant steroid implicated in treatment of scleroderma [10]. Another plant steroid is diosgenin, a component of Dioscorea plant that showed improvement of skin collagen [11]. Several of these plant steroids have been implicated to possess anti-inflammatory properties. However, it is not yet conclusive whether they mediate these properties through binding to GR. Thus, the aim of the current study is to apply molecular docking to comparatively screen these plant steroids against GR by orienting them in the GR-binding site, to choose the best candidate based on fitness score, pattern of binding, and energy values. In addition, the study is aimed to test the effect of the aforementioned plant steroids through *in vivo* studies on two animal models to investigate their potential anti-inflammatory properties, without adversely affecting the adrenal and/or thymus glands, which are major side effects of pharmacologically available glucocorticoids [5, 12].

## 2. Materials and Methods

### 2.1. Materials

GOLD Suite version 5.2.2 (license no. 4C72bqe56187) was purchased from Flexera software LLC (Belfast, UK). Croton oil and plant steroids, namely, glycyrrhetinic acid, guggulsterone, boswellic acid, withaferin A, and diosgenin, were purchased from Sigma-Aldrich Co. (St. Louis, MO, USA). Fluticasone was obtained as a generous gift from Sun Pharmaceutical Industries Ltd. (Vadodara, Gujarat, India). Diagnostic commercial enzyme-linked immunosorbent assay (ELISA) kits for the proinflammatory markers, tumor necrosis factor-*α* (TNF-*α*) and interleukin-6 (IL-6), were purchased from Krishgen Biosystems (Mumbai, India). All other reagents and chemicals used in this study were of analytical grade.

### 2.2. GR Protein and Ligand Preparation for Molecular Docking

Docking of plant steroids was carried out utilizing the glucocorticoid protein structure obtained from the Research Collaboratory for Structural Bioinformatics (RCSB) protein data bank (PDB ID 2V95) at a resolution of 1.96 Å. All ligands and water molecules were removed from the protein, with the addition of hydrogen. The high-potency GR ligand fluticasone was chosen as positive control, and its affinity to the receptor was compared with that of the plant steroids tested (glycyrrhetinic acid, guggulsterone, boswellic acid, withaferin A, and diosgenin), whose structures were drawn using SYBYL-X 1.2 software and energy minimized using the Tripos force field (Tripos/Certara, Princeton, NJ, USA).

### 2.3. Molecular Docking Using GOLD Suite Software

For *in silico* evaluation of protein-ligand interactions, a docking study was performed using GOLD docking suite version 5.2.2. The software used a genetic algorithm for docking and performed automated docking with fully cyclic ligand flexibility, partial cyclic ligand flexibility, and partial flexibility around the protein active site [13]. The docking process involved conformational search for a ligand, which compliments the binding site, with the aim of identifying the best binding pose into the protein active site, as previously described [14]. The interaction of the ligand protein complex involved hydrogen bonding and van der Walls interactions. The binding region for the docking study was defined as a 10 Å radius sphere centered on the active site. Default parameter values were used, and the complexes were submitted to 100,000 genetic algorithm runs using the GOLD Score fitness function [15].

### 2.4. Experimental Animals

Animal study protocols (IP/PCOL/FAC/22/2017/022) ethically followed the Research Ethics Committee, King Faisal University, which is in accordance with the National Committee of Bioethics (NCBE), KACST, Saudi Arabia, and the Institutional Animal Ethics Committee of Institute of Pharmacy, Nirma University, India. Swiss mice (28 ± 3 g) and SD rats (225 ± 25 g) were housed under controlled conditions of temperature (24 ± 2°C), humidity (55 ± 5%), and photo-schedule (12 h light and 12 h dark). Animals had free access to food and purified water *ad libitum* and were left to acclimatize for one week before starting the experiment.

### 2.5. Experimental Protocol of Croton Oil-Induced Ear Edema in Mice

Croton oil-induced ear edema in mice was performed to evaluate acute-phase inflammation [16]. The ear edema was induced by topical application of 20 *μ*l of croton oil 1% (*v*/*v*) in acetone on the inner surface of the right ear, while the left ear received 20 *μ*l of vehicle acetone. After 15 min, 25 *μ*g of glycyrrhetinic acid, guggulsterone, boswellic acid, withaferin A, diosgenin, or fluticasone was dissolved in 20 *μ*l of acetone and was topically applied on the right ear. The control group received 20 *μ*l of the vehicle acetone on the right ear. The ear edema was evaluated 6 h after croton oil application and was expressed as increase in ear weight (mg). The animals were sacrificed, and ear tissue samples (6 mm in diameter) were collected from the right and left ears of each animal by a metallic punch. The tissue samples were weighted, and edema was evaluated by the difference in weight between the right (inflamed) and left (noninflamed) ears. The results were expressed as the % inhibition.

### 2.6. Experimental Protocol of Cotton Pellet-Induced Granuloma Model in Rat

The cotton pellet-induced granuloma method, a well-known model to screen the anti-inflammatory activity in the chronic phase of inflammation, was performed as previously described [17]. Briefly, cotton pellets of 10 ± 1 mg were sterilized in an autoclave. Then, 25 *μ*g of glycyrrhetinic acid, guggulsterone, boswellic acid, withaferin A, diosgenin, or fluticasone dissolved in 40 *μ*l acetone was added on the autoclaved cotton pellets and dried aseptically. The negative control group received 40 *μ*l of vehicle acetone. The dried cotton pellets were aseptically implanted subcutaneously below the axilla of anesthetized rats. On day 8, blood samples were collected, and serum was separated for biochemical determination.

### 2.7. Assessment of Inhibition of Granuloma Formation as well as Adrenal and Thymus Gland Indices

At the end of the experiment, animals were killed, and cotton pellets were dissected out surgically and dried in a hot air oven at 60°C to a constant weight. The dried pellets were weighed and increment in the dry weight of pellets compared to control was taken as a measure of granuloma formation. The mean weight of the granuloma tissue in each group was recorded, and percentage inhibition was calculated by comparing the mean weight of the test group with the control group by using the following formula:

(1) $$\begin{array}{c} \text{Percent inhibition}=100*\left(\text{weight of control pellet}-\text{weight of test pellet}\right)/\text{weight of control pellet}. \end{array}$$

The adrenal and thymus glands of all rats were dissected out and weighed. The adrenal and thymus gland indices were expressed as the ratio (mg/g) of the adrenal and thymus glands, respectively, versus body weight [12].

### 2.8. Assessment of Serum Levels of IL-6 and TNF-*α* Using ELISA Technique

Serum from different groups of rats challenged with cotton pellet-induced granuloma was examined for the levels of IL-6 and TNF-*α* as proinflammatory markers using diagnostic commercial ELISA kits according to the manufacturer's instructions.

### 2.9. Statistical Analysis

All the values are expressed as mean ± standard error of mean (SEM). Statistical analysis was carried out using GraphPad Prism 5.0 version. Statistical significance between groups was tested using one-way ANOVA analysis followed by Tukey's multiple comparison test. Differences were considered statistically significant when *p* < 0.05.

## 3. Results

### 3.1. Molecular Docking with GR Protein

**Figure 1:**
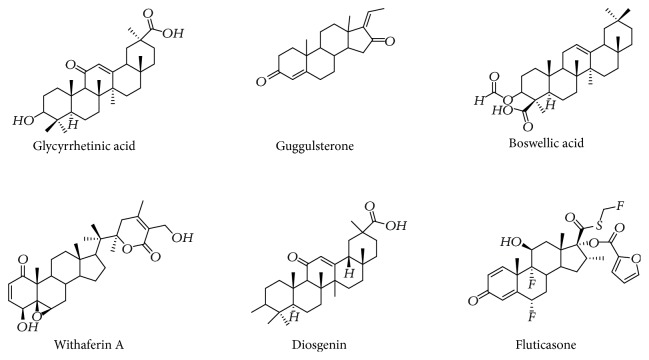
Chemical structures of plant steroids tested.

**Figure 2:**
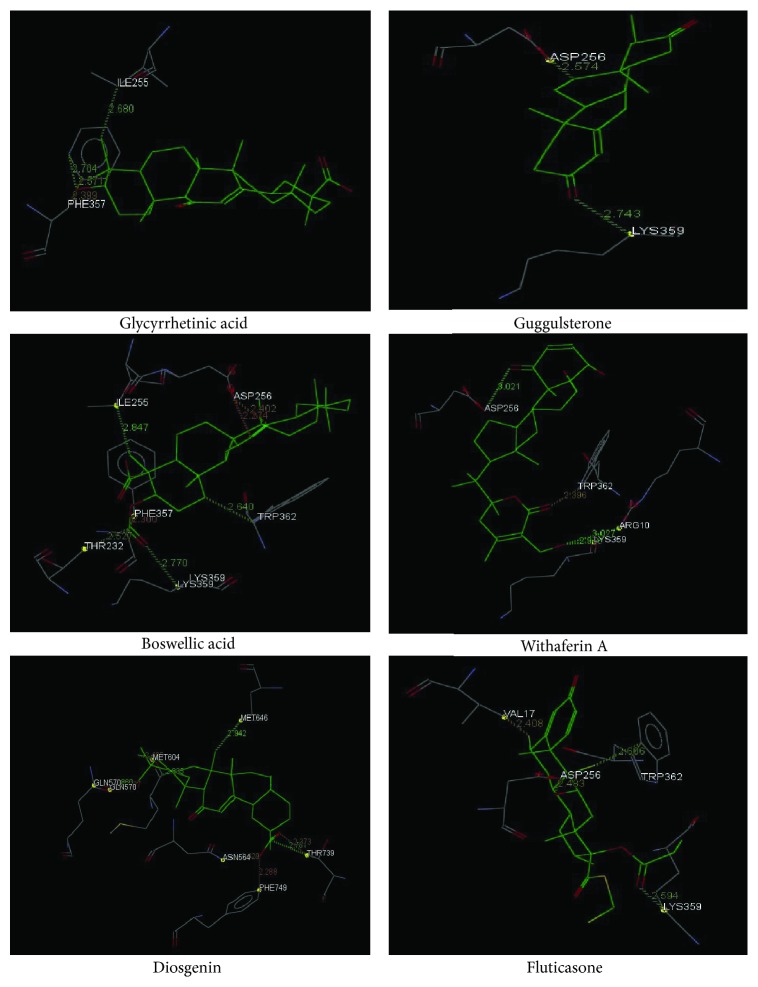
Binding interaction of plant steroids with the glucocorticoid receptor (PDB code: 2V95).

To compare the anti-inflammatory properties of plant steroids, we used the well-known GR ligand, fluticasone, to evaluate the binding modes and binding site interactions using GOLD docking suite version 5.2.2. The chemical structure of the tested plant steroids and fluticasone is shown in Figure 1. The docking scores and binding interactions with amino acids of all plant steroids and standard fluticasone are displayed in Table 1. Fluticasone displayed a good GOLD score and interaction with glucocorticoid amino acid residues (ASP 256, VAL 17, LYS 359, and TRP 362), out of which two were active site amino acids as shown in Figure 2. Diosgenin displayed a better GOLD score, which was comparable with fluticasone but was unable to show an interaction with active site amino acids. Similarly, glycyrrhetinic acid and guggulsterone revealed a good score, but displayed an interaction with only one active site amino acid PHE 357 and ASP 256, respectively. Boswellic acid showed a lower score compared to all plant steroids, but interacted with six amino acid residues, namely, LYS 359, ILE 255, THR 232, ASP 256, PHE 357, and TRP 362, out of which three were the active site amino acid residues. Withaferin A also showed an interaction with four amino acids, namely ASP 256, LYS 359, ARG 10, and TRP 362, out of which two were the active site amino acid residues involved in the interaction with a standard drug, fluticasone.

### 3.2. Effect on Croton Oil-Induced Ear Edema

Topical application of croton oil to the ears of mice resulted in a significant increase in the weight of the left ear as compared with the control right ear (*p* < 0.001). The percentage of inhibition of ear edema after topical application of plant steroids tested and fluticasone is summarized in Table 2 and shows that, except for glycyrrhetinic acid, all tested plant steroids significantly inhibited croton oil-induced ear edema. Interestingly, boswellic acid and withaferin A had comparable inhibition levels to those of the standard anti-inflammatory, fluticasone.

### 3.3. Effect on Percent Inhibition of Cotton Pellet-Induced Granuloma

Compared to the positive control, the effect of plant steroids and fluticasone on the increase in dry weight of implanted cotton pellets was evaluated after 8 days to assess their influence on the chronic phase of inflammation (Table 2). Apart from glycyrrhetinic acid and diosgenin, all the rest of the plant steroids tested significantly inhibited cotton pellet-induced granuloma, in an order of boswellic acid > withaferin A > guggulsterone. Surprisingly, the former had inhibition levels of cotton pellet-induced granuloma comparable to those caused by fluticasone.

### 3.4. Effect on Adrenal and Thymus Gland Indices

The effects of plant steroids and fluticasone on the adrenal and thymus gland indices were evaluated in cotton pellet-induced granuloma in rats (Table 3). Treatment of rats with topical fluticasone caused a significant decrease in the adrenal index as compared to diseased untreated rats. All tested plant steroids, however, did not affect the adrenal index. Similarly, the thymus index was significantly decreased by topical application of topical fluticasone, while all plant steroids tested did not affect the thymus index, with the exception of withaferin A that showed a significant reduction in the thymus index as compared to diseased untreated rats.

### 3.5. Effect on Serum IL-6 and TNF-*α* Levels

**Figure 3:**
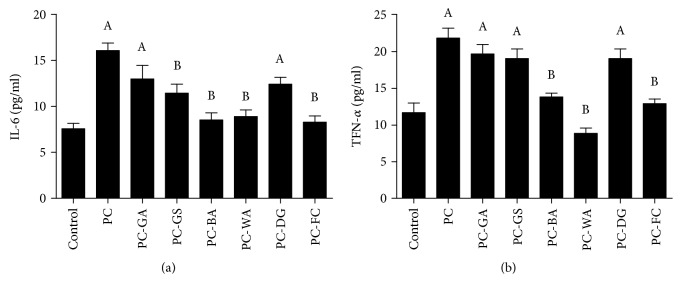
Effect of plant steroids and fluticasone on serum interleukin- (IL-) 6 and tumor necrosis factor- (TNF-) *α* levels in the cotton pellet-induced granuloma model. Serum IL-6 (a) and TNF-*α* (b) were evaluated using the ELISA technique. Values were expressed as mean ± SEM (*n* = 6) and analyzed by one-way ANOVA followed by Tukey's test. Results were considered significant if *p* values were less than 0.5. ^A^Significant compared to the control. ^B^Significant compared to the positive control (PC). PC-GA: PC treated with glycyrrhetinic acid; PC-GS: PC treated with guggulsterone; PC-BA: PC treated with boswellic acid; PC-WA: PC treated with withaferin A; PC-DG: PC treated with diosgenin; PC-FC: PC treated with fluticasone.

Using the ELISA technique, our results showed that the rats challenged with cotton pellet-induced granuloma had significantly elevated levels of the proinflammatory markers, IL-6 and TNF-*α*, as compared to normal control group (Figures 3(a) and 3(b), respectively). As expected, using the standard anti-inflammatory drug fluticasone caused a significant decrease in the serum level of IL-6 and TNF-*α*. In case of IL-6 (Figure 3(a)), only the plant steroids guggulsterone, boswellic acid, and withaferin significantly reduced serum IL-6 compared to the positive control, while glycyrrhetinic acid and diosgenin had no significant effect. On the other hand, in case of TNF-*α* (Figure 3(b)), boswellic acid and withaferin A only succeeded in decreasing the serum level of TNF-*α*, while glycyrrhetinic acid, guggulsterone, and diosgenin had no significant effect.

## 4. Discussion

Despite the vital role played by glucocorticoids in the medical field, their use is accompanied by a long list of adverse effects. Several previous attempts were made to discover novel agonists to GR which may be structurally similar to glucocorticoids and/or are accepted by GR as a ligand, which ideally have a similar treatment profile but fewer side effects than currently available glucocorticoids [18–20]. Botanical supplements containing plant steroids widely consumed worldwide might be considered a relatively safer alternative to glucocorticoids [6].

The GOLD docking results are reported in terms of the values of fitness, which means the higher the fitness the better the affinity of the ligand with protein [21]. In docking studies, apart from the docking score, the interaction of each ligand with the active site amino acids was also an important criterion. The 2V95 protein structure taken from the RCSB (PDB ID 2V95) displayed the interaction of a standard ligand, fluticasone, with active amino acids, namely, ASP 256, GLN 244, TRP 362, and PHE 357. Boswellic acid displayed a hydrogen bonding interaction with LYS 359 and short contacts with ILE 255, THR 232, TRP 362, ASP 256, and PHE 357 amino acids. Withaferin A showed a hydrogen bonding interaction with TRP 362, ARG 10, and LYS 359, as well as hydrophobic interactions with TRP 362 and ASP 256 amino acids. Despite that the GOLD score of glycyrrhetinic acid showed a higher affinity with GR, the binding mode showed that it interacted with GR with one active amino acid only, suggesting that glycyrrhetinic acid might not be the highest-affinity GR substrate among the compounds tested. Thus, a critical evaluation of the binding interaction of ligands with GR was done and suggested that boswellic acid and withaferin A displayed better binding modes with GR compared with the other tested ligands, where withaferin A interacted with two active site amino acids similar to those that fluticasone interacted with and boswellic acid interacted with three active site amino acid residues, which was even better than fluticasone. Previous *in silico* and/or *in vitro* studies suggested that these plant steroids might interact with GR, including glycyrrhetinic acid [22], guggulsterone [23], boswellic acid [24], withaferin A [25], and diosgenin [26]. However, none of the previous studies compared more than one plant steroids or referred their effect to a standard GR ligand as fluticasone.

The comparative effect of plant steroids tested on the inflammatory pathway was assessed by levels of IL-6 and TNF-*α* in serum of rats treated by subcutaneous implants of cotton pellets impregnated in the respective plant steroid. The previously reported explanation of the sequence of events in the inflammatory cascade among these two cytokines is complicated. The inflammatory trigger assault is thought to activate the NF-*κ*B pathway and causes its nuclear translocation, which causes upregulation of the proinflammatory cytokine TNF-*α*, while an increase in TNF-*α*, in turn, might further stimulate the nuclear translocation of NF-*κ*B, which causes the increase in expression of TNF-*α* itself as well as IL-6 [27], where the latter might have both proinflammatory and anti-inflammatory effects [28], acting as a feedback mechanism. In the current study, both cytokines were distinctly increased in the cotton pellet animal model, indicating an ongoing inflammatory process. Both boswellic acid and withaferin A succeeded in significantly reversing the increment in both IL-6 and TNF-*α* cytokines, suggesting having an anti-inflammatory mechanism. The anti-inflammatory properties have been previously suggested for boswellic acid through its interaction with NF-*κ*B [29]. Interestingly, guggulsterone had a differential effect on the two cytokines, as it decreased IL-6 but had no effect on TNF-*α*. Previous studies reported that guggulsterone had anti-inflammatory effects through interaction with NF-*κ*B, which was even higher in affinity, compared with boswellic acid [29], which is opposite to our results. Future studies using a wide range of doses of guggulsterone are necessary to elucidate this discrepancy.

The gross translation of the anti-inflammatory effect of tested plant steroids was confirmed on two animal models, namely, croton oil-induced ear edema in mice and cotton pellet granuloma in rats, which are well-established models of inflammation [30, 31]. Guggulsterone, boswellic acid, and withaferin A significantly improved the inflammatory process in both models, with boswellic acid and withaferin A having significance levels comparable to those of the standard glucocorticoid fluticasone in the croton oil-induced ear edema model, while boswellic acid only had significance levels comparable to fluticasone in a cotton pellet granuloma model. This is in line with previous reports indicating that boswellic acid may have beneficial anti-inflammatory and immunomodulatory properties [32].

Glycyrrhetinic acid, on the other hand, had no significant effect in either model, which is logical in view of its differential effect on serum cytokines. However, previous *in vitro* studies suggested that glycyrrhetinic acid probably had anti-inflammatory properties [33, 34], which contradict our results. It is possible that the concentration of glycyrrhetinic acid used in the current study was not high enough to show its beneficial effect in *in vivo* animal models. Interestingly, diosgenin improved the inflammation in the croton oil-induced ear edema in mouse model but not in the cotton pellet granuloma rat model. The previous study reported that diosgenin efficiently reduces skin inflammation in mice [35]. The difference in the anti-inflammatory effects of diosgenin among the two animal models may be explained by the different responses induced by croton oil and cotton pellet. Croton oil produces acute inflammation within three hours of application, due to the activation of phospholipase A2 and the release of arachidonic acid from the cell membrane [36]. On the other hand, the cotton pellet is a subacute model characterized by the proliferative phase of chronic inflammation lasting 3-6 days, during which the development of granuloma occurs due to proinflammatory mediator release, with subsequent altered vascular permeability and protein leakage initiating repair mechanism [37].

Atrophy of both thymus and adrenal glands is among the long list of side effects of conventional glucocorticoids, as reported in an *in vivo* study, in response to ciclesonide administration in rats [38]. Glucocorticoid therapy, even when given topically, produces adrenal insufficiency through triggering the hypothalamic/pituitary/adrenal axis which might continue for up to one year after termination of glucocorticoid therapy [5, 39]. Similarly in the thymus gland, the mechanism involved might include thymocyte apoptosis with cellular shrinkage and nuclear collapse leading to a decrease in the thymus index [38]. In the present study, application of the conventional glucocorticoid, fluticasone, caused a decrease in adrenal and thymus indices as compared to the positive control group. These systemic effects are consistent with the classic index of glucocorticoid actions on thymic and adrenal involution [12] and is probably because it has an extremely high lipophilicity, leading to a wide tissue distribution and longer plasma half-life [40]. Treatment with all tested plant steroids did not produce any significant effect on adrenal and thymus indices, except for withaferin A which considerably decreased the thymus weight, thus increasing its index, which was comparable to the fluticasone effect.

The ideal drug required to replace conventional glucocorticoids should be selective GR agonists that have the ability to separate therapeutic beneficial effects from unwanted side effects, by dissociation of transrepression from transactivation actions of the receptor activation, respectively [41]. Fluticasone does not exhibit preferential transrepression but might even possess higher potencies for transactivation than for transrepression, because of which it shows suppression of the hypothalamic/pituitary/adrenal axis [42]. On the other hand, boswellic acid, in the current study, showed the best binding profile with GR, even compared to fluticasone, which had anti-inflammatory beneficial effects comparable to fluticasone on both IL-6 and TNF-*α* and in both animal models tested, without inducing any adverse effect by suppression of the hypothalamic/pituitary/adrenal axis, indicated by a lack of harmful effect on adrenal and thymus indices, suggesting that boswellic acid shows preferential transrepression, favoring beneficial properties without adverse effects. Although the GR selectivity of boswellic acid and other plant steroids tested in the current study was done *in silico*, our results give a strong implication on such selectivity for future studies to explore a more detailed characterization of GR activation and signaling to support the anti-inflammatory effects of these natural products.

## 5. Conclusion

The current study compares several previously suggested plant steroids for their interaction with GR *in silico*. In addition, the anti-inflammatory properties of these compounds are compared *in vivo* using 2 different animal models. The ability of the tested plant steroids to cause less thymic/adrenal side effects is also tested. Our results showed that boswellic acid is a promising ligand for GR with anti-inflammatory properties comparable to conventional glucocorticoids and has even less side effects.

## Figures and Tables

**Table 1:** Docking of plant steroids and fluticasone with the glucocorticoid receptor.

Ligands	GOLD score	Bonding interaction with amino acids
Glycyrrhetinic acid	55.3	ILE 255, PHE 357
Guggulsterone	51.3	LYS 359, ASP 256
Boswellic acid	45.6	LYS 359, ILE 255, THR 232, ASP 256, PHE 357, TRP 362
Withaferin A	54.9	ASP 256, LYS 359, ARG 10, TRP 362
Diosgenin	53.3	GLN 570, MET 604, MET 646, ASN 564, THR 739, PHE 749
Fluticasone	61.8	ASP 256, VAL 17, LYS 359, TRP 362

**Table 2:** Effect of plant steroids and fluticasone on the percent of increase in ear weight in a croton oil-induced ear edema model and the granuloma weight in the cotton pellet-induced-granuloma model.

Ligands	Increase in ear weight (%)	Granuloma weight (mg)
PC	61 ± 7	127 ± 8
PC-GA	51 ± 3	109 ± 8
PC-GS	41±3^∗∗^	91±9^∗∗^
PC-BA	20±1^∗∗∗^	52±2^∗∗∗^
PC-WA	27±2^∗∗∗^	90±8^∗∗^
PC-DG	44 ± 4^∗^	101 ± 6
PC-FC	17±1^∗∗∗^	60±4^∗∗∗^

Values were expressed as mean ± SEM (*n* = 6) and analyzed by one-way ANOVA followed by Tukey's test. Results were considered significant if *p* values were less than 0.5. Significance was reported when *p* values were <0.05 (^∗^), <0.01 (^∗∗^), or <0.001 (^∗∗∗^) compared with the positive control (PC). PC-GA: PC treated with glycyrrhetinic acid; PC-GS: PC treated with guggulsterone; PC-BA: PC treated with boswellic acid; PC-WA: PC treated with withaferin A; PC-DG: PC treated with diosgenin; PC-FC: PC treated with fluticasone.

**Table 3:** Effect of plant steroids and fluticasone on the adrenal and thymus gland indices in the cotton pellet-induced granuloma model.

Ligands	Adrenal index	Thymus index
Control	3.1 ± 0.2^a^	22 ± 3^a^
PC	4.9 ± 0.4	39 ± 2
PC-GA	4.9 ± 0.2	39 ± 2
PC-GS	4.4 ± 0.3	37 ± 2
PC-BA	4.3 ± 0.2	34 ± 3
PC-WA	3.9 ± 0.1	29 ± 1^a^
PC-DG	4.3 ± 0.2	35 ± 3
PC-FC	3.3 ± 0.2^a^	26 ± 1^a^

Values were expressed as mean ± SEM (*n* = 6) and analyzed by one-way ANOVA followed by Tukey's test. Results were considered significant if *p* values were less than 0.5. Results were considered significant if *p* values were less than 0.5. ^a^Significant compared to positive control (PC). PC-GA: PC treated with glycyrrhetinic acid; PC-GS: PC treated with guggulsterone; PC-BA: PC treated with boswellic acid; PC-WA: PC treated with withaferin A; PC-DG: PC treated with diosgenin; PC-FC: PC treated with fluticasone.

## Data Availability

The data used to support the findings of this study are available from the corresponding author upon request.
